# Colombian Creole Horse: Frequency of oral and motor stereotypies

**DOI:** 10.14202/vetworld.2022.1113-1120

**Published:** 2022-04-27

**Authors:** Jhonny Alberto Buitrago Mejía, Jairo Alejandro Navarro Jaramillo, Natalia Uribe Corrales

**Affiliations:** 1 Department of Agricultural Sciences, Faculty of Veterinary Medicine, Lasallian University Corporation (Unilasallista), GIVET Research Group, Caldas, Antioquia, Colombia; 2 Department of Agricultural Sciences, Faculty of Veterinary Medicine, Remington University Corporation (Uniremington), Medellín, Antioquia, Colombia

**Keywords:** behaviours disorders, equines, stereotypies, welfare

## Abstract

**Background and Aim:**

The current stable housing of Colombian Creole Horses severely restricts the animals’ locomotion and natural behaviors. In addition, their feed consists of a combination of high-energy concentrates with considerably little forage which potentially leads to locomotor or oral stereotypies. This study aimed to report the frequency of locomotor and oral stereotypies in Colombian Creole Horses in Girardota (Antioquia, Colombia) and associated risk factors.

**Materials and Methods:**

A prospective cross-sectional study was conducted from 2019 to 2020, in which 102 stabled horses aged 28 months and older participated. A questionnaire was developed to collect information on the horses’ daily barn routines. The horses were observed twice a day for 2 h for 3 consecutive days to record information related to stereotypy behaviors. The Fisher’s exact test and the Mann–Whitney U test were utilized for data analyses. Associations were considered statistically significant at p<0.05.

**Results:**

Among the horses evaluated, 32.35% presented at least one stereotyped behavior. The most common was crib-biting (i.e., cribbing), with 17.65% exhibiting this behavior. Age, weight, gender, type of feeding, visual contact between horses, and natural lighting were all associated with oral stereotypies. Crib-biting was most common in young horses (U=1.36, p≤0.05), wind-sucking was more common in lighter weight animals (U=1.45, p=0.01), and lip-smacking was more common in stallions (χ^2^=9.10, p≤0.01). It is noteworthy that their feeding diet included bran, molasses, and gopher. Horses that did not have visual contact with other horses and those that did not have natural lighting were associated with pica (χ^2^=9.52, p≤0.02; χ^2^=3.72, p≤0.05; and χ^2^=3.72, p≤0.05, respectively). Of locomotor stereotypies, kicking the wall was significant in young animals (U=1.54, p=0.03) and walking in circles in lactating mares (χ^2^=13.20, p≤0.02).

**Conclusion:**

Housing conditions in this study were found to have several risk factors affecting horses that exhibit stereotypic behaviors, and all these factors resulted in a higher frequency of stereotypies. Establishing risk factors for the presentation of abnormal behaviors allows for the implementation of better management practices in the production systems of the Creole Colombian Horse and will help improve their overall welfare.

## Introduction

Current housing conditions of domestic horses compared with a natural situation are very different. This is especially true of Colombian Creole Horses, which are frequently housed in environments that often severely restrict their locomotion. They are also often fed a combination of high-energy concentrates with relatively little forage, usually only twice daily. This results in the horses being food deprived for relatively long periods and having inadequate stall infrastructure and bedding material [1-7].

Therefore, even if current housing environments are better at facilitating the handling of horses by owners and veterinarians, they often limit the social interactions of horses with their peers [6,8]. In addition, they can result in alterations in their feeding and physical exercise schedule [9]. Due to these conditions, stabled horses may display forms of locomotor or oral stereotypies more frequently than horses managed under other conditions.

The most accepted definition of stereotypy is a behavior pattern that is repetitive and invariant with no apparent goal or function [10,11]. However, Mason [12] defined stereotypy as repetitive behavior induced by frustration, repeated coping attempts, or central nervous system dysfunction. These stereotypies can reduce the conception rate in mares and contribute to other health problems [13,14]. In addition, stereotypies can reduce the economic value of horses and are one of the most important indicators of long-term welfare problems in these animals [15].

According to some studies, there is a genetic predisposition toward stereotypic display that could be associated with temperament [1,16]. However, there are other risk factors, including age [17,18], gender [19], type of bedding [20], feeding frequency [21], usage of concentrate feed [22], lack of access to pasture [5,23], and restriction of free movement [24]; all of which can increase the risk of manifesting locomotor or oral stereotypies.

Behavioral problems in horses can be categorized into three groups [25]:

- Oral stereotypies, such as crib-biting, wind-sucking, wood chewing, teeth grinding, self-mutilation, coprophagy, lip-smacking, and pica (the abnormal desire to eat substances not customarily eaten, i.e., non-food items).
- Locomotor stereotypies include weaving, stall circling, head nodding, and wall kicking.
- Social problems, including training problems and phobic responses.

One of Colombia’s most popular breeds of horses is the Colombian Creole Horse, used for recreational riding and exhibitions. These horses are characterized by having a nervous and energetic temperament and are usually kept in barns differentiated by specific training regimes. All these factors are predispose to developing stereotypies [26]. Despite this, few studies in Colombia have provided information on stereotypies, especially in this type of horse [26,27].

Therefore, the objective of this study was to report the frequency of locomotor and oral stereotypies in Colombian Creole Horses in Girardota (Antioquia, Colombia) and associated risk factors.

## Materials and Methods

### Ethical approval and Informed consent

This study was framed under Colombian law 8430 of 1993, law 84 of 1989 and law 1774 of 2016. It was considered a study without risk, and the animal’s rights were respected. Likewise, the project had the ethics endorsement (No. 04) of the Uniremington University Corporation as stated in act 005/2018. All the owners signed the informed consent before starting the observation of the animals.

### Study type, period, and location

**Figure-1:**
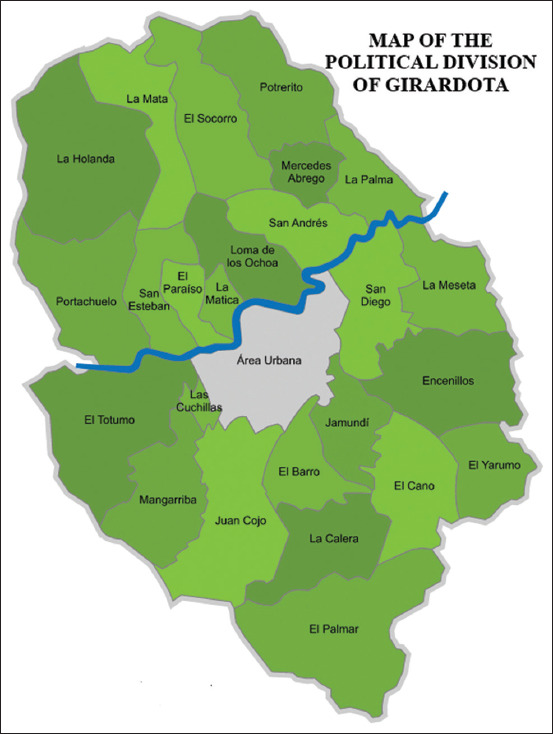
Map of Girardota, Antioquia-Colombia [29].

A prospective cross-sectional study was carried out in 2019-2020. A non-probabilistic sampling was carried out with 102 equines distributed to 13 barns in Girardota (Antioquia, Colombia). Girardota is a town in Colombia, located in Antioquia. It borders the towns of San Pedro de los Milagros, Donmatías, Barbosa, San Vicente, Guarne and Copacabana. Its geographical coordinates are 6° 22’37” N 75 ° 26’46” W. This town has an area of 82.56 km², of which 3.07 km² are urban land, and 79.49 km² are rural land. Its average temperature is 22°C and an average height of 1.425 m above sea level [28] (Figure-1), [29].

### Population

This study included Colombian Creole Horses aged more than 28 months and which were managed in barns full time. Horses that presented pre-existing pathologies were undergoing environmental enrichment or were undergoing medical treatment were excluded from the study.

### Data collection

The owners signed informed consent forms, and then veterinarians performed a general physical examination to check the health status of each horse. The same veterinarian always performed this activity. Subsequently, a questionnaire related to the characteristics and handling of the horse was conducted, and, finally, the animal was observed over a time period as described in this section.

### Questionnaire

A questionnaire was created to record the horses’ daily barn routine. Various data, including feeding programs, training regimen, and stall size, were collected during an interview with the horse’s owner or barn manager.

### Observation

The horses were observed twice a day for 2 h for 3 consecutive days. The observation of the horses was conducted 45 min before feeding, 30 min during their feeding time, and 45 min after they had finished feeding. An ethogram suggested by Tadich *et al*. [19] was completed. Information related to stereotypies manifested by the animals and the time dedicated to each behavior was recorded.

### Statistical analysis

The data were analyzed with the Stata^®^ program version 15 (https://www.stata.com/stata15/), license number: 301506348331. A frequency analysis was conducted in which proportions were determined for qualitative variables, and the means and their respective standard deviations were determined for quantitative variables. To investigate the relationship between animal-specific and environmental variables and the presence or absence of abnormal behavior, the Fisher’s exact test and Mann-Whitney U test were performed. The Kolmogorov–Smirnov test was conducted to determine normal distribution. Associations were considered statistically significant at p≤0.05.

## Results

One hundred two horses were evaluated in 13 barns in Girardota. The horses had an average age of 5.80±3.58 years, a mean weight of 325.77±50.18 kg, and 59.80% of horses were mares. Among the males, 22% were geldings. It was found that the average assessed body condition on a scale from 1 to 9 was 7.00±1. The other analyzed characteristics of the animals are shown in Table-1.

**Table 1:** Horses and barns characteristics.

Variable	Category	Absolutely frequency (n=102)	Percentage
Mares (n=61)	Empty	45	44.12
	Pregnant	11	10.78
	Lactation	4	3.92
	Weaned	1	0.98
Males (n=41)	Stallion	32	29.41
	Gelding	9	22.00
Gaits	Canter	39	38.24
	Trot and gallop	37	36.27
	Canter and gallop	16	15.69
	Smooth steeper	10	9.80
Age	From 2 to 5-years-old	61	59.80
	From 6 to 10-years-old	31	30.39
	From 11 to 15-years-old	8	7.84
	More than 15-years-old	2	1.97
Incisor wear	Yes	24	23.13
	No	78	76.57
Spicules	Yes	19	18.63
	No	83	81.37
Barn location layout	Parallel	52	50.98
	Linear	42	41.17
	Horseshoe	6	5.88
	Internal	2	1.96
Feeder/drinker distribution	Side to side	64	62.74
	Opposite corners	36	35.29
	Together	2	1.96
Contact between horses	Yes	92	90.20
	No	10	9.80
Bedding	Wood chip	96	94.12
	Sawdust	6	5.88
Natural lighting	Yes	92	90.20
	No	10	9.80

The horses’ feed was found to consist of 49.02% concentrate, 27.45% cut grass, 17.65% molasses and corncob, and 5.88% bran. Regarding the handling of the horses, there was no evidence of shouting or harsh treatment from the stable workers toward the horses. In addition, it was found that the workers changed the water an average of 2 times a day, and the horses performed an average of 36.96±13.48 min of physical activity daily.

The drinker and feeder boxes were observed to be made of plastic of 77.0% of the time and were made of cement 23.0% of the time. On average, the total area of each box was 4.91±1.47 m^2^ (Table-1).

**Figure-2:**
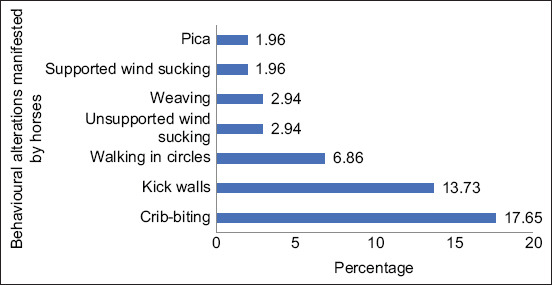
Motor and oral stereotypies manifested by the Colombian Creole Horses.

Of the stereotypies observed, 32.35% of the horses presented at least one, with the most common being crib-biting (17.65%; Figure-2). Similarly, it can be seen that wind-sucking was the behavior that had the longest duration (Table-2).

**Table 2:** Duration time of different behavioral alterations.

Alteration	Behavioral alterations	The average duration in minutes (Standard deviation)
Oral	Supported wind sucking	19.33 (10.96)
	Unsupported wind sucking	6.51 (2.22)
	Crib-biting	4.79 (4.59)
	Lip-smacking	0.81 (0.44)
Motor	Weaving	9.71 (8.38)
	Kick walls	3.59 (3.71)
	Walking in circles	2.78 (3.14)
	Digging	2.5 (2.12)

### Oral stereotypies

Age, weight, gender, type of feeding, visual contact between horses, natural lighting, distribution of feeder and drinker boxes, and tactile contact between horses were associated with oral stereotypies. Crib-biting was more common in young horses (U=1.36, p≤0.05); wind-sucking was more common in lighter animals when feeders and drinkers were close together, and when the animals did not have tactile contact (U=1.45, p=0.01; χ^2^=15.94, p≤0.00; χ^2^=5.20, and p≤0.02, respectively); lip-smacking was more usual in stallions (χ^2^=9.10, p≤0.01). Finally, horses fed with bran, molasses, and gopher, which did not have visual contact with other horses, and which did not have natural lighting, were associated with pica (χ^2^=9.52, p≤0.02; χ^2^=3.72, p≤0.05; and χ^2^=3.72, p≤0.05, respectively) (Tables-3 and 4).

**Table 3:** Association between stereotypies and animal characteristics.

Variable	Category	Motor Stereotypies						Oral Stereotypies									
	
		Kick the wall		Weaving		Walk in circles		Crib-biting		Wind sucking with support		Wind sucking without support		Lip-smacking		Pica	
							
		Median	U (p-value)	Median	U (p-value)	Median	U (p-value)	Median	U (p-value)	Median	U (p-value)	Median	U (p-value)	Median	U (p-value)	Median	U (p-value)
Age	No	5.00	**1.54 (0.03)** *	5.00	0.47 (0.67)	5.00	0.42 (0.75)	5.00	**1.36 (0.05)** *	5.00	0.62 (0.34)	5.00	0.75 (0.12)	5.00	0.12 (0.94)	5.00	0.09 (0.95)
	Yes	3.00		3.00		5.00		4.00		3.50		9.00		3.50		6.00	
Weight (Kg)	No	320.00	0.34 (0.77)	320.00	0.98 (0.06)	320.00	0.65 (0.20)	307.00	0.16 (0.4)	320.00	**1.45 (0.01)** *	320.00	0.92 (0.06)	320.00	0.78 (0.28)	320.00	0.10 (0.96)
	Yes	322.00		268.00		300.00		347.50		240.50		368.00		354.00		324.00	
Exercise per day (minutes)	No	30.00	0.45 (0.71)	30.00	0.11 (0.95)	30.00	0.81 (0.13)	30.00	0.45 (0.92)	30.00	0.32 (0.83)	30.00	0.19 (0.95)	30.00	0.12 (0.94)	30.00	0.31 (0.74)
	Yes	30.00		30.00		30.00		30.00		35.00		30.00		30.00		40.00	

U: U Mann–Whitney Test.

* Associations statistically significant

**Table 4:** Association between oral stereotypies with animal and environment variables.

Variable	Category	Cri-biting OR (95% CI)	F (p- value)	Supported wind sucking OR (95% CI)	F (p- value)	Unsupported wind sucking OR (95% CI)	F (p- value)	Lip- smacking OR (95% CI)	F (p-value)	Pica OR (95% CI)	F (p-value)
Gender	Mare	1.06 (0.89-0.95)	1.47 (0.68)	1.08 (0.82-1.15)	0.56 (0.90)	1.04 (1.00-1.09)	4.76 (0.18)	1.07 (0.98-1.09)	6.21 (0.10)	1.08 (0.82-1.15)	0.56 (0.90)
	Stallion	1.10 (0.75-1.23)		1.18 (1.02-1.29)		1.01 (0.67-1.45)		1.21 (0.80-9.13)		1.18 (1.02-1.29)	
	Gelding	1		1		1		1		1	
Reproductive status	Empty	0.91 (0.84-1.10)	2.29 (0.80)	1.00 (0.95-1.06)	0.85 (0.97)	0.94 (0.86-1.06)	4.76 (0.44)	0.98 (0.91-1.10)	6.21 (0.28)	1.09 (0.98-1.12)	0.85 (0.97)
	Pregnant	1.06 (0.89-0.95)		1.03 (0.98-1.07)		1.03 (0.98-1.07)		0.91 (0.84-1.10)		0.98 (0.94-1.02)	
	Lactation	1.03 (0.98-1.07)		1.01 (0.98-1.12)		0.98 (0.91-1.10)		1.03 (0.98-1.07)		0.92 (0.84-1.09)	
	Weaned	0.91 (0.84-1.10)		1.08 (0.82-1.15)		1.08 (0.82-1.15)		1.08 (0.82-1.15)		0.95 (0.91-1.07)	
	Stallion	1.13 (0.99-1.25)		1.15 (0.98-1.21)		1.32 (0.56-1.89)		0.2 (0.016-2.42)		1.03 (0.98-1.11)	
	Gelding	1		1		1		1		1	
Food offered	Concentrate	1.81 (0.18-18.14)	1.55 (0.67)	0.2 (0.016-2.42)	1.89 (0.59)	0.58 (0.03-9.67)	0.80 (0.84)	1.81 (0.18-18.14)	4.89 (0.17)	0.80 (0.75-0.90)	**9.52 (0.02)** *
	Bran	1.04 (1.00-1.09)		1.03 (0.98-1.07)		0.91 (0.84-1.10)		1.01 (0.67-1.45)		0.76 (0.71-0.86)	
	Cut grass	1.06 (0.89-0.95)		1.04 (1.00-1.09)		1.23 (0.86-1.84)		1.06 (0.89-0.95)		0.78 (0.84-0.95)	
	Bran. molasses and gopher	1		1		1		1		**1**	
Barn location layout	Internal	0.58 (0.03-9.67)	6.56 (0.08)	1.03 (0.98-1.07)	0.19 (0.97)	0.91 (0.84-1.10)	0.43 (0.93)	**2.34 (1.40-3.84)**	**13.70 (0.00)** *	1.03 (0.98-1.07)	0.19 (0.97)
	Horseshoe	1.03 (0.98-1.07)		1.04 (1.00-1.09)		1.03 (0.98-1.07)		0.91 (0.84-1.10)		1.01 (0.98-1.12)	
	Linear	1.01 (0.98-1.12)		1.05 (0.88-1.09)		1.23 (0.86-1.84)		0.98 (0.90-1.13)		1.07 (0.86-1.13)	
	Parallel	1		1		1		1		1	
Feeder/drinker distribution	Opposite corners	1.02 (0.89-1.21)	1.47 (0.47)	0.2 (0.016-2.42)	1.21 (0.27)	1.08 (0.82-1.15)	**15.94 (0.00)** *	1.05 (0.88-1.09)	0.44 (0.80)	0.2 (0.016-2.42)	1.21 (0.54)
	Together	0.98 (0.84-1.17)		0.58 (0.03-9.67)		**2.11 (1.42-3.65)**		1.03 (0.97-1.22)		0.58 (0.03-9.67)	
	Side to side	1		1		1		1		1	
Visual contact among horses	No	2.04 (0.24-17.20)	0.44 (0.50)	1.02 (0.99-1.05)	0.22 (0.63)	0.2 (0.016-2.42)	1.91 (0.16)	1.04 (1.00-1.09)	0.44 (0.50)	1.18 (1.02-1.29)	**3.72 (0.05)** *
	Yes	1		1		1		1		1	
Tactile contact among horses	No	2.38 (0.72-7.85)	2.11 (0.14)	1.03 (0.98-1.07)	1.21 (0.27)	**7.89 (1.01-16.04)**	**5.20 (0.02)** *	1.81 (0.18-18.14)	0.26 (0.60)	0.58 (0.03-9.67)	0.14 (0.70)
	Yes	1		1		1		1		1	
Natural lighting	No	2.04 (0.24-17.20)	0.44 (0.50)	1.02 (0.99-1.05)	0.22 (0.63)	0.2 (0.016-2.42)	1.91 (0.16)	1.04 (1.00-1.09)	0.44 (0.50)	**1.18 (1.02-1.29)**	**3.72 (0.05)** *
	Yes	1		1		1		1		1	

F=The Fisher’s test.

* Associations statistically significant, OR=Odds ratio, CI=Confidence interval, bold values=Category of the variable that is different

### Locomotor stereotypies

The behavior of wall kicking was found to be common in young animals (U=1.54, p=0.03), and walking in circles was common in lactating mares (χ^2^=13.20, p≤0.02) (Tables-3 and 5).

**Table 5:** Association between motor stereotypies with animal and environment variables.

Variable	Category	Kick walls OR (CI 95%)	F (p-value)	Weaving OR (CI 95%)	F (p-value)	Walking in circles OR (CI 95%)	F (p-value)
Gender	Mare	1.01 (0.54-1.52)	2.91 (0.40)	1.05 (0.88-1.09)	0.41 (0.93)	0.22 (0.016-2.42)	0.94 (0.81)
	Stallion	1.20 (0.68-1.12)		1.03 (0.97-1.22)		0.54 (0.03-9.67)	
	Gelding	1		1		1	
Reproductive status	Empty	0.98 (0.94-1.02)	3.73 (0.58)	1.13 (0.99-1.25)	7.61 (0.17)	1.01 (0.85-1.02)	**13.20 (0.02)** *
	Pregnant	1.03 (0.98-1.07)		1.03 (0.98-1.11)		1.04 (1.00-1.09)	
	Lactation	1.04 (1.00-1.09)		0.92 (0.84-1.09)		**2.94 (1.65-4.94)**	
	Weaned	0.80 (0.75-0.90)		1.08 (0.82-1.15)		0.80 (0.75-0.90)	
	Stallion	1.08 (0.82-1.15)		0.76 (0.71-0.86)		1.02 (0.95-1.06)	
	Gelding	1		1		1	
Food offered	Concentrate	1.05 (0.88-1.09)	4.98 (0.17)	0791 (0.84-1.10)	3.21 (0.35)	0.31 (0.07-1.40)	1.52 (0.67)
	Bran	1.08 (0.82-1.15)		1.43 (0.98-1.77)		1.04 (1.00-1.09)	
	Cut grass	0.2 (0.016-2.42)		1.23 (0.86-1.84)		1.08 (0.82-1.15)	
	Bran. molasses and gopher	1		1		1	
Barn location layout	Internal	1.04 (0.78-1.21)	1.58 (0.66)	0.98 (0.91-1.10)	0.43 (0.93)	1.14 (0.91-1.23)	6.36 (0.09)
	Horseshoe	1.04 (1.00-1.09)		1.08 (0.82-1.15)		1.04 (1.00-1.09)	
	Linear	0.95 (0.45-1.16)		0.92 (0.84-1.09)		1.32 (0.84-1.95)	
	Parallel	1		1		1	
Feeder/drinker distribution	Opposite corners	1.02 (0.89-1.21)	2.90 (0.23)	0.80 (0.75-0.90)	0.07 80.96)	1.01 (0.98-1.12)	1.71 (0.42)
	Together	0.98 (0.84-1.17)		0.76 (0.71-0.86)		1.04 (1.00-1.09)	
	Side to side	1		1		1	
Visual contact among horses	No	0.31 (0.07-1.40)	2.47 (0.11)	1.03 (0.99-1.07)	0.33 (0.56)	0.62 (0.06-5.81)	0.17 (0.52)
	Yes	1		1		1	
Tactile contact among horses	No	0.38 (0.12-1.32)	2.74 (0.09)	0.28 (0.02-3.26)	1.14 (0.28)	1.52 (0.28-8.27)	0.24 (0.62)
	Yes	1		1		1	
Natural lighting	No	0.31 (0.07-1.40)	2.47 (0.11)	1.03 (0.99-1.07)	0.33 (0.56)	0.62 (0.06-5.81)	0.17 (0.52)
	Yes	1	1	1	

F=The Fisher’s test.

* Associations statistically significant, OR=Odds ratio, CI=Confidence interval, bold values=Category of the variable that is different

## Discussion

In the current study, oral and locomotor stereotypies had the same percentage of presentation, with both being observed in 24.51% of the animals. Some studies have reported that horses kept in barns have a higher frequency of locomotor stereotypies, whereas those that do not have *ad libitum* access to forage will manifest oral stereotypies. Both conditions were found to be present in the population evaluated [11,24,27].

The most common observed stereotypy was crib-biting. This behavior has been reported as highly prevalent in horses from Europe, Canada, and the United States, although their frequencies are lower than seen in this study [30].

The results show a higher frequency of stereotypy among Creole Horses from Chile and English Thoroughbreds. Studies of these breeds found stereotypies observed in 4-11% of Chilean Creole Horses [10,18] and 6-12% of English Thoroughbred horses [19,31,32]. Nonetheless, the frequency of abnormal behaviors found here was lower than reported previously in Colombia, Costa Rica, and Chile, where authors found 65%, 48.4%, and 56.9% of horses exhibiting these behaviors, respectively [26,33,34].

Some risk factors, such as the breed type, have been reported. In Chile, differences were found in the distribution of stereotypies, and in Chilean Creole Horses, a higher frequency of walking in circles was reported, followed by crib-biting [18,35]. In English Thoroughbred horses, the stereotypy most frequently seen was wind-sucking, followed by stall circling and weaving [31,32]. In Fino Chilote, the most frequent stereotype observed was weaving, followed by wind-sucking [34].

It has been reported that crib-biting has been associated with social isolation and lack of grazing [36,37] and is seen as a more common postprandial behavior in horses with little fiber in their diet [38,39]. A higher incidence of crib-biting behavior has also been reported in foals fed concentrates after weaning than foals that did not receive concentrates [39]. One possible explanation for crib-biting, the most frequent stereotypy, is that in horses that are kept permanently housed, their ability to express natural grazing behaviors has been limited, thus resulting in stereotypies [40].

Some previous studies have not found associations between stereotypies and gender or age [31,35], yet in this study, different associations were found between gender, age, and oral or locomotor stereotypies. These differences could be the result of differences in the sample populations of these studies compared to the population involved in this study, confounding the data. Specifically, most of the [1,6,19] were conducted on young racehorses with the same handling regardless of animal gender. Related to gender, this study found an association with the presentation of lip-smacking mainly in stallions to be similar to other reports, which have also shown that the risk is more significant in male horses working in competitions [18,19].

The characteristics of feeding type, level of visual contact between horses, and access to natural lighting were all found to be factors related to the presentation of pica. This behavior was present in the animals fed with bran, molasses, and gopher and with among horses that do not have visual contact with other horses. This finding corresponds to other studies in which it has been reported that isolation can increase the risk of developing abnormal behaviors due to lack of socialization [18]. Locomotor stereotypies observed were similar to findings of other reports and included young animals that kicked the walls more frequently and lactating mares that were found to more frequently walk in circles [32].

Although this study followed a recommended methodology for the evaluation of behavior, it had the limitation of not being able to make observations during 24 consecutive h. Despite this limitation, it provides information that allows for the estimation of the behavioral alterations manifested by horses related to mealtime, as well as to the time invested in those behavioral alterations.

## Conclusion

This study found that horses’ housing conditions were related to several risk factors in the study area of Girardota, Columbia. These included no access to pasture, no ad libitum feeding, and the use of feed concentrates. All these factors resulted in a higher frequency of oral stereotypies. In addition, prolonged confinement in individual horse stalls was found to be positively correlated with stereotypies. Establishing risk factors for the presentation of stereotypies will allow the implementation of good management practices in the production systems of the Colombian Creole Horse and will thereby improve their welfare.

## Authors’ Contributions

JABM: Conceptualization. JANJ: Data collection. NUC: Formal analysis. JABM, JANJ, and NUC: Investigation. NUC: Methodology. JABM, JANJ, and NUC: Writing original draft. NUC: Writing – review and editing. All authors have read and approved the final manuscript.
